# Factor structure and psychometric validation of the antenatal exercise knowledge, attitudes, and practices questionnaire: A secondary analysis of cross-sectional data from Saudi Arabian pregnant women

**DOI:** 10.1097/MD.0000000000051300

**Published:** 2026-10-23

**Authors:** Abdullah Hussain Alzahrani, Wael Alghamdi

**Affiliations:** a Department of Health Rehabilitation, College of Applied Medical Sciences at Shaqra, Shaqra University, Shaqra, Saudi Arabia; b Faculty of Nursing, Al-Baha University, Saudi Arabia.

**Keywords:** antenatal exercise, factor analysis, knowledge-attitude-practice, measurement invariance, psychometric validation, reliability, Saudi Arabia

## Abstract

Assessing pregnant women’s knowledge, attitudes, and practices (KAP) vis-à-vis antenatal exercise is essential for designing targeted interventions that improve maternal and fetal outcomes. The previously published KAP questionnaire has been used in Saudi Arabia, but its latent structures and psychometric properties require thorough examination. The aim of this study was to perform an extended exploratory factor analysis and detailed psychometric evaluation of the antenatal exercise KAP questionnaire, including assessment of factorability, dimensionality, reliability, and item-level psychometric performance. We conducted a secondary analysis on item-level data from 118 pregnant women. In addition to principal-axis factoring with Varimax rotation, we applied parallel analysis for factor retention and assessed item communalities, cross-loadings, and measurement invariance across primiparous and multiparous subgroups. Internal consistency was evaluated using Cronbach’s α, item-total correlations, and McDonald ω. All scales met adequacy criteria (knowledge Kaiser–Meyer–Olkin [KMO] = 0.756; attitude KMO = 0.674; practice KMO = 0.631; Bartlett *P* < .001). Parallel analyses and scree plots suggested unidimensionality for each domain. The first factor explained 78.6% (knowledge), 94.1% (attitude), and 95.8% (practice) of the variance. Communalities were uniformly high (>0.50), and no substantial cross-loadings (>0.30) were observed. Cronbach’s α (knowledge = 0.991; attitude = 0.996; practice = 0.990) and McDonald ω (0.989–0.995) corroborated high reliability. Measurement invariance testing indicated configural and metric invariance across parity groups. The antenatal exercise KAP questionnaire showed preliminary evidence of a unidimensional structure and high internal consistency in this sample. Given the modest sample size relative to the number of items, particularly in the knowledge domain, confirmation in larger independent samples is warranted.

## 1. Introduction

Physical activity during pregnancy is widely endorsed by international health organizations, including the World Health Organization,^[1]^ the American College of Obstetricians and Gynecologists,^[2]^ and the Canadian Society for Exercise Physiology,^[3]^ as a cornerstone of prenatal care due to its documented benefits on maternal and fetal health outcomes. These benefits include reduced risk of gestational diabetes mellitus, pre-eclampsia, excessive weight gain, and depressive symptoms, as well as enhanced cardiovascular fitness and improved postpartum recovery.^[4]^ Despite these clear recommendations, global adherence to antenatal exercise guidelines remains suboptimal, particularly in Middle Eastern contexts where cultural beliefs, limited awareness, and misconceptions about exercise safety during pregnancy pose significant barriers.^[5–7]^

In Saudi Arabia, rapid socioeconomic change, urbanization, and shifting lifestyle patterns have contributed to increased rates of obesity and sedentary behavior among women of reproductive age, underscoring the urgent need for evidence-based interventions that promote safe, culturally appropriate physical activity during pregnancy.^[8]^ To inform such interventions, valid and reliable measurement of pregnant women’s knowledge, attitudes, and practices (KAP) vis-à-vis antenatal exercise is essential. The antenatal exercise KAP questionnaire (which comprises a 37-item knowledge scale, a 16-item attitude scale, and a 6-item practice scale) was developed and piloted in a number of studies on Saudi women, demonstrating acceptable face and content validity, as well as preliminary internal consistency.^[9–11]^ However, the tool’s latent structure and comprehensive psychometric properties have not been rigorously examined.

Exploratory factor analysis (EFA) is a fundamental method for uncovering latent constructs within multi-item instruments and guiding scale refinement by identifying redundant or poorly performing items.^[12]^ Furthermore, reliability indices such as Cronbach’s α provide an estimate of internal consistency, whereas alternative metrics such as McDonald omega (ω) can offer a more nuanced view of scale reliability when distributions are skewed or item loadings vary.^[13]^ Measurement invariance testing^[14]^ further ensures that the instrument measures constructs equivalently across key subpopulations, which is critical for comparing scores between primiparous and multiparous women, whose previous pregnancy experiences may influence their understanding and behaviors related to exercise.

Building on previous research by Alghamdi et al,^[11]^ this study aims to thoroughly evaluate the psychometric properties of the antenatal exercise KAP questionnaire in a Saudi Arabian sample. Specifically, this study investigates the underlying structure of each domain using EFA (supported by parallel analysis and scree-plot inspection), assesses internal consistency (using Cronbach’s α, McDonald omega, and item-total correlations), examines score distributions for ceiling and floor effects, and tests measurement invariance across parity groups to ensure the questionnaire’s relevance for diverse pregnant populations. This comprehensive evaluation is expected to provide essential insights into the questionnaire’s validity, reliability, and potential for refinement, thereby strengthening its usefulness in both research and clinical antenatal care settings, particularly in Saudi Arabia.

## 2. Methods

### 2.1. Study design and participants

This study involved a secondary psychometric re-analysis of previously collected and published data from Alghamdi.^[11]^ The original data were collected between January and June 2022 at a tertiary care hospital in Riyadh, Saudi Arabia. The questionnaire items were adapted from a previously validated instrument that has been used in studies conducted across different countries.^[15,16]^ The original sample comprised 118 pregnant women aged 21 to 55 years, with gestational ages ranging from 1 to 9 months, who were carrying singleton pregnancies and were able to read Arabic. Women with obstetric complications contraindicating physical activity or chronic cardiovascular disease were excluded. Ethical approval for the study was obtained from the Ethical Review Board of Shaqra University (ERC_SU_S_202500108). The present analysis used the existing deidentified dataset exclusively for additional psychometric evaluation. Informed consent obtained during the original study covered the subsequent secondary analysis of the collected data.

### 2.2. Data collection and instrument

Participants completed an antenatal exercise KAP questionnaire, which includes 3 domains. The knowledge section consists of 37 true/false items evaluating the participant’s awareness of the benefits, safety considerations, and types of antenatal exercise. The attitude section includes 16 statements rated on a 5-point Likert scale, which were subsequently rescored to a 0 to 1 scale to reflect the degree of positive attitudes. The practice section contains 6 items capturing self-reported frequency of antenatal exercise behaviors, also standardized to a 0 to 1 scale. In addition to KAP data, baseline demographic and clinical characteristics such as age, parity, educational attainment, and body mass index were collected.

### 2.3. Data analysis

Data were processed and analyzed using Statistical Package for the Social Sciences version 27 (IBM Corp.). For items with missing responses (<5% per item), pairwise deletion was applied for factor analysis, whereas expectation–maximization methods were used to calculate reliability estimates. Item distributions were evaluated for normality by inspecting skewness and kurtosis values, with acceptable thresholds set at ±2. Attitude items that were negatively worded were reverse-coded to ensure consistent scoring direction across the scale.

### 2.4. Exploratory factor analysis

EFA was conducted separately for each domain of the questionnaire. Before extraction, assumptions for factorability were confirmed using the Kaiser–Meyer–Olkin (KMO) measures of sampling adequacy (threshold ≥0.60) and Bartlett test of sphericity (*P* < .05). In addition, anti-image correlations were reviewed to ensure that all individual items had measures of sampling adequacy ≥0.50. Factors were extracted using principal-axis factoring, retaining components with eigenvalues greater-than-one. Parallel analysis using 1000 Monte Carlo simulations further guided factor retention decisions. Both orthogonal (Varimax) and oblique (Direct Oblimin) rotations were applied to assess factor structure and potential correlations between factors, with factor intercorrelation coefficients below 0.30 considered evidence of independence. Items were retained if they demonstrated primary factor loadings ≥0.40 and secondary loadings ≤0.30, indicating a clean and interpretable structure.

Given the sample size of 118 participants relative to the 37 items in the knowledge domain (approximately 3.2 participants per item), the EFA findings were considered exploratory. Interpretation, therefore, also considered empirical indicators of factorability and solution quality, including the KMO statistic, Bartlett test, communalities, factor loadings, and parallel analysis, rather than relying solely on a subjects-to-item ratio.

### 2.5. Reliability and distributional analyses

Internal consistency of each domain was evaluated using Cronbach’s α, with a target threshold of ≥0.70, and McDonald omega was also calculated to provide a more robust reliability estimate. Additional metrics included corrected item-total correlations (target ≥0.30) and communalities (target ≥0.50) to assess item contribution and shared variance. Ceiling and floor effects were assessed by calculating the proportions of participants who scored at the extremes of each domain; a threshold of >15% was considered indicative of potential concern in score distribution.

Given the possibility that very high reliability coefficients may reflect item redundancy, item coding and reverse scoring were rechecked, and inter-item relationships and item-total correlations were examined for evidence of near-duplicate or excessively homogeneous items.

### 2.6. Measurement invariance

To evaluate whether the factor structure was consistent across subgroups, a multi-group confirmatory factor analysis (CFA) was performed using IBM Statistical Package for the Social Sciences Amos, version 27.0 (IBM Corp.), with parity (primiparous vs multiparous) as the grouping variable. Configural and metric invariance were tested sequentially. Model fit was assessed using standard indices: Comparative Fit Index (CFI) values of ≥0.90 and root mean square error of approximation (RMSEA) values of ≤0.08 indicated acceptable fit. A change in CFI (ΔCFI) of <0.01 between models was considered evidence of invariance.

## 3. Results

The analytic sample comprised 118 pregnant women, which has been discussed in more detail previously.^[11]^

### 3.1. Assumptions for a factorability test

Assessment of sampling adequacy and sphericity confirmed that each domain was appropriate for factor analysis. KMO measures were 0.756 for the knowledge domain, 0.674 for attitude, and 0.631 for practice, all exceeding the minimum threshold of 0.60. Bartlett tests of sphericity were highly significant in all cases (knowledge: χ^2^[666] = 54,113.36; attitude: χ^2^[120] = 28,548.39; practice: χ^2^[15] = 8174.98; all *P* <.001), indicating that correlations among items were sufficient to proceed (Table 1).

**Table 1:** Sampling adequacy and sphericity tests by domain.

Domain	KMO	Bartlett χ^2^ (df)	*P*
Knowledge	0.756	54,113.36 (666)	<.001
Attitude	0.674	28,548.39 (120)	<.001
Practice	0.631	8174.98 (15)	<.001

The table presents KMO measures of sampling adequacy and Bartlett test of sphericity for each questionnaire domain, confirming the appropriateness of the data for factor analysis.

KMO = Kaiser–Meyer–Olkin.

### 3.2. Exploratory factor analysis

EFA, informed by parallel analysis and inspection of scree plots, supported a unidimensional structure for each scale. In the knowledge domain, a single factor with an eigenvalue of 29.09 emerged, explaining 78.6% of variance; item loadings ranged from 0.62 to 0.89, with communalities between 0.53 and 0.92. The attitude domain likewise yielded 1 factor (eigenvalue = 15.06) accounting for 94.1% of variance, with loadings from 0.72 to 0.95 and communalities from 0.52 to 0.90. Similarly, the practice domain demonstrated a single factor (eigenvalue = 5.75) explaining 95.8% of variance, with item loadings spanning 0.75 to 0.98 and communalities between 0.57 and 0.96 (Table 2).

**Table 2:** Exploratory factor analysis outcomes for each domain.

Domain	Eigenvalue	% Variance explained	Loading range	Communality range
Knowledge	29.09	78.6%	0.62–0.89	0.53–0.92
Attitude	15.06	94.1%	0.72–0.95	0.52–0.90
Practice	5.75	95.8%	0.75–0.98	0.57–0.96

This table details the eigenvalues, percentages of variance explained, factor loading ranges, and communality ranges for the single-factor solutions identified in each questionnaire domain, illustrating the strength and coherence of the latent constructs.

Inspection of the inter-item correlation matrices did not identify near-perfect correlations indicative of severe collinearity, although generally strong inter-item correlations may partly explain the large proportion of variance accounted for by the first factor.

### 3.3. Reliability and distributional analysis outcomes

All scales exhibited very high internal consistency, with coefficients exceeding conventional thresholds and potentially indicating substantial overlap among some items. Cronbach’s α coefficients were 0.991 for knowledge, 0.996 for attitude, and 0.990 for practice, whereas McDonald omega values were 0.993, 0.995, and 0.989, respectively. Corrected item-total correlations exceeded 0.55 across all items, and no ceiling or floor effects exceeded 9%, indicating robust score dispersion (Table 3).

**Table 3:** Internal consistency reliability coefficients by domain.

Domain	Cronbach’s α	McDonald ω
Knowledge	0.991	0.993
Attitude	0.996	0.995
Practice	0.990	0.989

This table reports Cronbach’s α and McDonald ω for each domain, demonstrating the exceptional internal consistency and reliability of the knowledge, attitude, and practice scales.

### 3.4. Measurement invariance

Multi-group CFA (Table 4) in analysis of moment structures demonstrated configural invariance across primiparous (n = 10) and multiparous (n = 108) women (CFI = 0.962; RMSEA = 0.041). Metric invariance was also supported, with the metric model showing acceptable fit (CFI = 0.957; RMSEA = 0.044), exceeding the prespecified CFI threshold of 0.90, and only a minimal change from the configural model (ΔCFI = 0.005).

**Table 4:** Multigroup invariance testing results.

Model	CFI	RMSEA	ΔCFI
Configural	0.962	0.041	—
Metric (loadings)	0.957	0.044	0.005

This table shows fit indices for configural and metric invariance models in AMOS, indicating the stability of the factor structure across primiparous and multiparous subgroups.

AMOS = analysis of moment structures, CFI = comparative fit index, RMSEA = root mean square error of approximation.

## 4. Discussion

The present study offers a multifaceted psychometric evaluation of the antenatal exercise KAP questionnaire, providing preliminary support for its unidimensional structure, excellent reliability, and measurement equivalence in a sample of Saudi pregnant women. Here, we aimed to incorporate advanced factor retention methods such as parallel analysis, leverage alternative reliability metrics such as McDonald omega, and investigate measurement invariance across subgroups, thus attempting to provide a more comprehensive assessment of the tool’s psychometric robustness than was previously available.

Our findings align with broader KAP research in related health domains, where single-factor structures often emerge following thorough validation procedures. For example, unidimensional constructs have been identified in instruments measuring diabetes self-management, such as the Diabetes Self-Management Questionnaire, which demonstrated a coherent single-factor solution and strong psychometric properties in a sample of type 1 and type 2 diabetes patients.^[17]^ Similarly, unidimensionality has been confirmed in breastfeeding self-efficacy measures, notably the Breastfeeding Self-Efficacy Scale-Short Form, which exhibited excellent fit for a single-factor model among postpartum women in a confirmatory analysis,^[18]^ reinforcing the theoretical expectation that beliefs and behaviors around specific health practices coalesce around core underlying dimensions. Nonetheless, antenatal exercise questionnaires have rarely undergone such extensive scrutiny, underscoring the novelty of our work and its contribution to the literature by confirming that pregnant women’s exercise-related knowledge, attitudes, and behaviors do indeed form cohesive constructs amenable to total-score interpretation.

In practical terms, the validated questionnaire offers clinicians, public health practitioners, and policymakers a concise yet sensitive instrument for assessing KAP profiles related to antenatal exercise.^[19]^ Healthcare providers can use total and item-level scores to identify common misconceptions or attitudinal barriers that may impede exercise adoption, and thus customize counseling and exercise prescriptions accordingly.^[19]^ At the population level, aggregated KAP data can inform the development of culturally appropriate guidelines and targeted intervention campaigns, ensuring that educational resources are directed toward areas of greatest need. The minimal ceiling and floor effects observed further guarantee that the instrument captures a broad range of responses, enhancing its utility for both baseline assessments and monitoring change following interventions.

In addition, this new analysis sheds light on specific item performance, revealing which content areas may benefit from greater emphasis in educational programs.^[20]^ Items with relatively lower factor loadings point to topics where respondents demonstrate less clarity or confidence, such as safety considerations or self-efficacy.^[21]^ These insights can guide the creation of targeted learning modules (delivered via digital platforms, group workshops, or printed materials) to address identified knowledge gaps and reinforce positive attitudes and behaviors.

Methodologically, this study illustrates the value of combining multiple psychometric techniques. Parallel analysis provided a rigorous basis for factor retention beyond the traditional eigenvalue-greater-than-one rule, whereas confirmatory invariance testing ensured that the instrument measures KAP equivalently across primiparous and multiparous women.^[22,23]^ Future research should build upon these methods by evaluating the predictive validity of the questionnaire, linking baseline KAP scores to objective exercise behaviors and key maternal-fetal outcomes, such as gestational weight gain or glycemic control. Longitudinal studies assessing the sensitivity of the instrument to change in response to educational interventions would also enhance its applicability in clinical trials and program evaluations. The exceptionally high α and ω coefficients should nevertheless be interpreted cautiously. Reliability values approaching 1.0 may indicate considerable overlap or redundancy among items rather than proportionately greater measurement quality. Although coding and scoring should be verified to exclude computational artifacts, the high coefficients observed here also support future item-reduction analyses to identify potentially redundant items while preserving adequate construct coverage.

Furthermore, integrating the validated questionnaire into digital health solutions represents an exciting frontier. Embedding the tool within mobile or web-based platforms could facilitate real-time data collection, automated scoring, and personalized feedback. Decision-support algorithms could generate tailored exercise recommendations and behavior-change prompts based on individual KAP profiles, thereby optimizing engagement and adherence to antenatal physical activity regimens.^[24]^

In acknowledging the limitations of this discussion, we recognize that while structural and reliability aspects have been rigorously addressed, the linkage between KAP scores and actual behavioral and clinical outcomes is still to be established. An additional limitation is the modest sample size relative to the number of questionnaire items, particularly for the 37-item knowledge domain, for which the participant-to-item ratio was approximately 3.2:1. Although the KMO value, communalities, factor loadings, and parallel analysis supported factorability and a coherent solution, the relatively small sample may limit the stability and reproducibility of the extracted factor structure. Accordingly, these findings should be regarded as preliminary and confirmed using CFA in a larger independent sample. Furthermore, although we confirmed parity-based invariance, additional work is needed to evaluate the questionnaire’s equivalence across other demographic groups, including variations in age, education level, and prepregnancy activity patterns. The measurement invariance findings should be interpreted cautiously because of the small and markedly unbalanced subgroup sizes (primiparous, n = 10; multiparous, n = 108), which limit statistical power and may affect the stability of parameter estimates. Accordingly, these findings should be considered exploratory and require confirmation in larger, more balanced samples. Despite these considerations, the extended validation presented here supports the antenatal exercise KAP questionnaire being a concise, reliable measure capable of guiding targeted interventions and advancing maternal health promotion in diverse care settings.

## 5. Conclusion

The antenatal exercise KAP questionnaire showed preliminary evidence of a unidimensional factor structure, high internal consistency, and measurement invariance among Saudi pregnant women. These findings support the potential utility of the questionnaire, although the factor structure should be confirmed in a larger independent sample, particularly given the relatively low participant-to-item ratio for the knowledge domain.

The availability of such a validated tool has significant potential for both clinical and public health applications, particularly in identifying educational needs and tailoring antenatal interventions that promote maternal physical activity. To enhance its generalizability and cross-cultural applicability, future research should aim to refine the scale through CFA, examine its predictive validity against behavioral outcomes, and validate it in more diverse populations across different regions and healthcare settings.

## Acknowledgments

The authors would like to thank the Deanship of Scientific Research at Shaqra University for supporting this work.

## Author contributions

**Conceptualization:** Abdullah Hussain Alzahrani, Wael Alghamdi.

**Data curation:** Abdullah Hussain Alzahrani, Wael Alghamdi.

**Formal analysis:** Abdullah Hussain Alzahrani, Wael Alghamdi.

**Investigation:** Abdullah Hussain Alzahrani, Wael Alghamdi.

**Methodology:** Abdullah Hussain Alzahrani, Wael Alghamdi.

**Project administration:** Abdullah Hussain Alzahrani, Wael Alghamdi.

**Resources:** Abdullah Hussain Alzahrani, Wael Alghamdi.

**Software:** Abdullah Hussain Alzahrani, Wael Alghamdi.

**Supervision:** Abdullah Hussain Alzahrani, Wael Alghamdi.

**Validation:** Abdullah Hussain Alzahrani, Wael Alghamdi.

**Visualization:** Abdullah Hussain Alzahrani, Wael Alghamdi.

**Writing – original draft:** Abdullah Hussain Alzahrani, Wael Alghamdi.

**Writing – review & editing:** Abdullah Hussain Alzahrani, Wael Alghamdi.

**Funding acquisition:** Wael Alghamdi.
